# Prevalence of *Salmonella* Isolates and Their Distribution Based on Whole-Genome Sequence in a Chicken Slaughterhouse in Jiangsu, China

**DOI:** 10.3389/fvets.2020.00029

**Published:** 2020-02-21

**Authors:** Dan Gu, Zhenyu Wang, Yuqi Tian, Xilong Kang, Chuang Meng, Xiang Chen, Zhiming Pan, Xinan Jiao

**Affiliations:** ^1^Jiangsu Co-innovation Center for Prevention and Control of Important Animal Infectious Diseases and Zoonoses, Yangzhou University, Yangzhou, China; ^2^Jiangsu Key Laboratory of Zoonosis, Yangzhou University, Yangzhou, China; ^3^Key Laboratory of Prevention and Control of Biological Hazard Factors (Animal Origin) for Agrifood Safety and Quality, Ministry of Agriculture of China, Yangzhou University, Yangzhou, China; ^4^Joint International Research Laboratory of Agriculture and Agri-Product Safety of the Ministry of Education, Yangzhou University, Yangzhou, China

**Keywords:** *Salmonella*, whole-genome sequencing, serovars, MLST, antimicrobial resistance

## Abstract

*Salmonella* has been known as the most important foodborne pathogen, which can infect humans via consuming contaminated food. Chicken meat has been known as an important vehicle to transmit *Salmonella* by the food supply chain. This study determined the prevalence, antimicrobial resistance, and genetic characteristics of *Salmonella* at different chicken slaughtering stages in East China. In total, 114 out of 200 (57%) samples were *Salmonella* positive, while *Salmonella* contamination was gradually increasing from the scalding and unhairing stage (17.5%) to the subdividing stage (70%) throughout the slaughtering. Whole-genome sequencing (WGS) was then performed to analyze the serotype, antimicrobial resistance gene profiles, and genetic relationship of all *Salmonella* isolates. The most common serotypes were *S*. Kentucky (51/114, 44.7%) and *S*. Enteritidis (37/114, 32.5%), which were distributed throughout the four slaughtering stages, and were also identified in the corresponding environments. The multilocus sequence typing (MLST) analysis revealed that seven sequence types (STs) were occupied by six different serotypes, respectively. Only *S*. Kentucky had two STs, ST314 was the predominant ST shared by 50 isolates, while the ST198 has 1 isolate. The antimicrobial resistance gene analysis demonstrated that most of the strains belonging to *S*. Kentucky (39/51, 76.5%) and *S*. Indiana (15, 100%) contained over five groups of antimicrobial resistance genes. Based on the core genome analysis, 50 *S*. Kentucky isolates were genetically identical, indicating that one *S*. Kentucky strain with the same genetic background was prevalent in the chicken slaughtering line. Although 37 *S*. Enteritidis isolates only had three different antimicrobial resistance gene profiles, the core genome sequence analysis subtyped these *S*. Enteritidis isolates into five different clusters, which revealed the diverse genetic background of *S*. Enteritidis in the slaughterhouse. The antimicrobial resistance phenotypes were consistent with the presence of the corresponding resistance genes of *S*. Kentucky and *S*. Enteritidis, including *tetA, floR, blaTEM-1B, strA/B, sul1*/*sul2*, and *gyrA* (D87Y). Our study observed a high prevalence of *Salmonella* in the chicken slaughter line and identified the slaughtering environment as a main source of causing *Salmonella* cross-contamination during chicken slaughtering. Further studies will be needed to limit the transmission of *Salmonella* in the slaughterhouse.

## Background

*Salmonella* is an important foodborne pathogen causing gastroenteritis in humans and animals (1, 2). In USA, 46,623 cases of culture-confirmed *Salmonella* infection were reported from 53 states and regional public health laboratories in 2016, in which summer was the high-incidence season (3). In Europe, 91,662 confirmed human salmonellosis cases were reported by all member states in 2017 (4). In China, ~70–80% of foodborne pathogenic outbreaks are caused by *Salmonella*, and most of them are derived from animal-origin food products (5).

*Salmonella* are prevalent in domestic animals such as poultry, pigs, and cattle, and can be transmitted through the food chain by the animal-origin food products (6–8). Slaughter is considered as an important step causing *Salmonella* contamination in meat products (6, 7). A study demonstrated that the total isolation rate of *Salmonella* was 34.0% in a pig slaughterhouse in Hainan, China, and cross-contamination was also observed during the slaughtering process (9). In northern Italy, *Salmonella* was found in 12.3 and 11.2% of carcass samples from two pig slaughterhouses, respectively, indicating the potential transmission of *Salmonella* from slaughterhouse to retail meat (10). However, limited studies were conducted on the prevalence of *Salmonella* in chicken slaughterhouse in China.

The prevalent study had shown that the most common serotypes in *Salmonella* human cases in Europe were *S*. Enteritidis, *S*. Typhimurium, I 4,[5],12:i:-, *S*. Infantis, and *S*. Newport, while in the US, the most common serotypes were *S*. Enteritidis, *S*. Newport, *S*. Typhimurium, *S*. Javiana, and I 4,[5],12:i:- (3, 4). In China, *S*. Typhimurium were identified as the most common serotypes from humans followed by *S*. Enteritidis, *S*. Derby, and *S*. Indiana (11). Another research showed that the MLST of *S*. Enteritidis identified from humans was ST11 (12). The most common serotypes from the chicken were *S*. Enteritidis, followed by *S*. Indiana and *S*. Typhimurium, while the predominant MLST types were ST11, ST17, and ST19 in Shandong province of China (13, 14).

This study was to evaluate the distribution of *Salmonella* in different slaughtering stages/environments in a chicken slaughterhouse in summer and autumn. We selected four key slaughtering stages for sampling including scalding and dehairing, evisceration, pre-cooling, and subdividing. Based on whole-genome sequencing (WGS), we further analyzed the serotype, MLST, and antimicrobial resistance genes of all *Salmonella* isolates and evaluate the occurrence and distribution of *Salmonella* at different slaughtering steps and environments.

## Methods

### Sample Collection and *Salmonella* Isolated

A total of 160 carcass swab samples and 40 environment samples were collected from a poultry slaughterhouse during August and October, 2018, in Jiangsu, China. Twenty carcass samples and five environment samples were collected at four different slaughtering steps including scalding and dehairing, evisceration, pre-cooling, and subdividing.

The isolation of *Salmonella* was performed as previously described (9). In brief, 100 ml of buffered peptone water (BPW) was added to cotton swab samples and incubated at 37°C overnight. Then, 1 ml of enriched BPW suspension was transferred to Rappaport-Vassiliadis R10 broth (RVR10), incubated at 42°C for 24–48 h, and further streaked on XLT4 agar plate and incubated at 37°C for 24 h for *Salmonella* selection. Presumptive *Salmonella* colonies were confirmed as *Salmonella* by PCR with the presence of the *stn* gene. The PCR program of *stn* gene was performed as previously described (15) the PCR results are shown in Figure S1.

### WGS, Assembly, and Analysis

The genomic DNA of all *Salmonella* isolates were extracted by TIAN amp Bacteria DNA Kit (Tiangen, Beijing, China). All the genomes were fragment with an insertion size of 500 bp to construct the library, and the NEB Next Ultra DNA Library Prey Kit for illumina (NEB, Beverly, MA, USA) was used to generate sequencing libraries followed by the manufacturer's recommendation, and the WGS of libraries was performed by illumina platform Hiseq 2500. SPAdes version 3.10.0 was used to assemble the reads into contigs (16), and the information is shown in Table S1. The serotypes were analyzed by *Salmonella In Silico* Typing Resource (SISTR) (17). The multilocus sequence typing (MLST) of all isolates was conducted by Seemann MLST database (https://cge.cbs.dtu.dk/services/MLST/) (18). Antimicrobial resistance genes of each isolate were analyzed by ResFinder 3.2 database (https://cge.cbs.dtu.dk/services/ResFinder/) (19). WGS data of all *Salmonella* isolates were submitted to the European Nucleotide Archive with the accession number PRJEB34962.

### Antimicrobial Susceptibility Testing (AST)

AST was based on the Clinical and Laboratory Standards Institute (CLSI 2018). The agar dilution method was performed to determine the minimal inhibitory concentration (MIC) of the *Salmonella* isolates to the antimicrobial drugs. The test antibiotics included tetracycline, chloramphenicol, ciprofloxacin, ampicillin, cefazolin, cefotaxime, nalidixic acid, trimethoprim-sulfamethoxazole, and streptomycin. *Escherichia coli* ATCC 25922 was used for quality control strain.

### Statistical Analysis

The proportions of *Salmonella* in different slaughtering steps of the two visits were based on ANOVA comparisons with SPSS statistical package (SPSS Inc., Chicago, USA). Statistical significance was set at *P* ≤ 0.05.

## Results

### Prevalence of *Salmonella* in a Chicken Slaughterhouse

A total of 114 (57.0%) *Salmonella* strains were isolated from 160 carcass swab samples and 40 environment samples at different slaughtering steps (Table 1). The *Salmonella* prevalence rate at different slaughtering steps showed no significant difference between the two visits (*P* = 0.737). The highest prevalence of *Salmonella* was observed at the subdividing link stage, in which 70% (28/40) of the samples were *Salmonella* positive, followed by pre-cooling with 65.0% (26/40) of positive samples and evisceration with 60.0% (24/40) of positive samples, respectively. The lowest prevalence of *Salmonella* was at the scalding and unhairing stage, in which only 17.5% (7/40) of samples were *Salmonella* positive. The result demonstrated that the prevalence of *Salmonella* in this slaughterhouse showed an increasing trend through the sequential processes. In addition, 72.5% (29/40) of the environment samples were *Salmonella* positive, and the prevalence rates showed no significant difference between the two visits, indicating the environment as an important arena for the cross-contamination of *Salmonella*.

**Table 1:** Prevalence of *Salmonella* isolated from carcass swab samples and environmental samples.

	**Sample size per visit**	**Visit 1**		**Visit 2**		**Total ratio %**	**Serotype**	**Number**		**MLST**
		**Number**	**Ratio %**	**Number**	**Ratio %**			**Visit 1**	**Visit 2**	
Scalding and Unhairing	20	0	0.0	7	35.0	17.5	*S*. Kentucky	-	4	ST314
							*S*. Enteritidis	-	3	ST11
Evisceration	20	17	85.0	7	35.0	60.0	*S*. Kentucky	5	4	ST314
							*S*. Enteritidis	4	3	ST11
							*S*. Indiana	7	-	ST14
							*S*. Corvallis	1	-	ST1541
Pre-cooling	20	13	65.0	13	65.0	70.0	*S*. Kentucky	5	6	ST314
							*S*. Enteritidis	4	5	ST11
							*S*. Indiana	3	-	ST14
							*S*. Corvallis	1	-	ST1541
							I 4,[5],12:i:-	-	2	ST34
Subdividing	20	14	70.0	14	70.0	70.0	*S*. Kentucky	5	7	ST314
							*S*. Kentucky	1		ST198
							*S*. Enteritidis	3	7	ST11
							*S*. Indiana	3	-	ST14
							*S*. Corvallis	2	-	ST1541
Environment	20	16	80.0	13	65.0	72.5	*S*. Kentucky	8	6	ST314
							*S*. Enteritidis	2	6	ST11
							*S*. Indiana	2	-	ST14
							*S*. Corvallis	3	-	ST1541
							I 4,[5],12:i:-	-	1	ST34
							*S*. Hadar	1	-	ST33
**Total**	100	60	60.0	54	54.0	57.0	**Total**	60	54	

**Figure 1:**
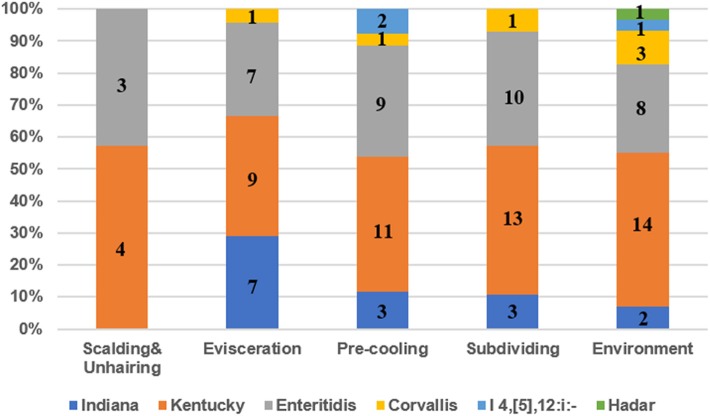
The prevalence of serotypes of *Salmonella* isolates from different slaughtering stages and environments. Numbers represent the isolate numbers of different *Salmonella* serotypes in different steps.

Six different serotypes were identified from 114 *Salmonella* isolates based on WGS analysis (Table 1 and Table S2). The most prevalent serotype was *S*. Kentucky (44.7%, 51/114), followed by *S*. Enteritidis (32.5%, 37/114), *S*. Indiana (13.0%, 15/114), *S*. Corvallis (6.1%, 7/114), *Salmonella* I 4,[5],12:i:- (2.6%, 3/114), and *S*. Hadar (0.9%, 1/114). Both *S*. Kentucky and *S*. Enteritidis were identified in the two visits. *S*. Indiana, *S*. Corvallis, and *S*. Hadar only appeared in the first visit, while *Salmonella* I 4,[5],12:i:- only appeared in the second visit. *S*. Kentucky and *S*. Enteritidis appeared in all four slaughtering steps and their related environments during the two visits, indicating the persistence of these two serotypes in the slaughtering line (Figure 1). Moreover, *S*. Indiana and *S*. Corvallis were found after the evisceration step for the first visits, indicating that contamination by these two serotypes may occur at this stage. *S*. Hadar was only observed in the slaughtering environment, indicating the low cross-contamination possibility of this serotype. MLST analysis showed that these 114 *Salmonella* isolates into seven STs (Table 1). Fifty out of 51 *S*. Kentucky strains were ST314 with only one isolate from ST198. All 37 *S*. Enteritidis isolates belonged to ST11, while all 15 *S*. Indiana isolates belonged to ST14. By correlating the STs to serotypes of all isolates, we observed a close relationship of these two typing results. These results indicate that one ST corresponds to one serotype, but different isolates belonging to one serotype may share multiple STs, which is consistent with previous studies (20).

Among the 13 plasmids identified in the 114 isolates, the most prevalent plasmid was IncX1 (55/114, 48.3%), followed by IncR (43/114, 37.7%), IncFIB(S)/IncFII(S) (32/114, 28.1%), IncQ1 (12/114, 10.5%), and CoI440I (9/114, 7.9%) (Table S3). In addition, the IncX1 plasmid was predominant in *S*. Enteritidis isolates, while IncR was the most prevalent plasmid in *S*. Kentucky isolates.

### Antimicrobial Analysis

In total, 10 different groups of antibiotic resistance genes (ARG) were detected from 106 out of 114 *Salmonella* genomes. All of the ARGs and their frequency of occurrence in *Salmonella* isolates are listed in Table S2. 54.39% (*n* = 62) of isolates displayed ARGs related to the resistance to at least five groups of antibiotics, and 24.56% (*n* = 28) of isolates contained at least 8of the 10 groups of ARG. All 15 *S*. Indiana isolates, 3 *Salmonella* I 4,[5],12:i:- isolates, and 39 of 51 *S*. Kentucky isolates contained more than five classes of ARGs. Our results demonstrated a high prevalence of multidrug resistance *Salmonella* in the slaughter line and the related environments. The antimicrobial resistance genes were sporadically identified in the isolates, which are all listed in Table S4.

The resistant phenotype of quinolone was known to regulate by point mutant in the quinolone resistance-determining regions (QRDRs) of *gyrA, gyrB, parC*, and *parE* (21), and the plasmid-mediated quinolone resistance genes (22). The mutation of QRDRs in the *Salmonella* isolates is shown in Table S5. Interestingly, we also observed that different mutations in QRDRs were closely related to serotypes. All *S*. Indiana isolates, *S*. Hadar isolates, 35 of 37 *S*. Enteritidis, and 1 of 51 *S*. Kentucky isolates contained point mutations at *gyrA*, indicating that these isolates may be resistant to nalidixic acid and ciprofloxacin. Four quinolone-resistance-associated genes were identified in these isolates, in which *qnrB6* (33.33%, 38/114) was the most prevalent, followed by *qnrS1* (7.02%, 8/114), *oqxB* (2.63%, 3/114), and *oqxA* (1.75%, 2/114). Fifty of 51 *S*. Kentucky strains did not have the mutation of *gyrA*, whereas quinolone-resistance gene *qnrB6* was detected in 35 isolates.

### Genomic Analysis of *S*. Kentucky Isolates

**Figure 2:**
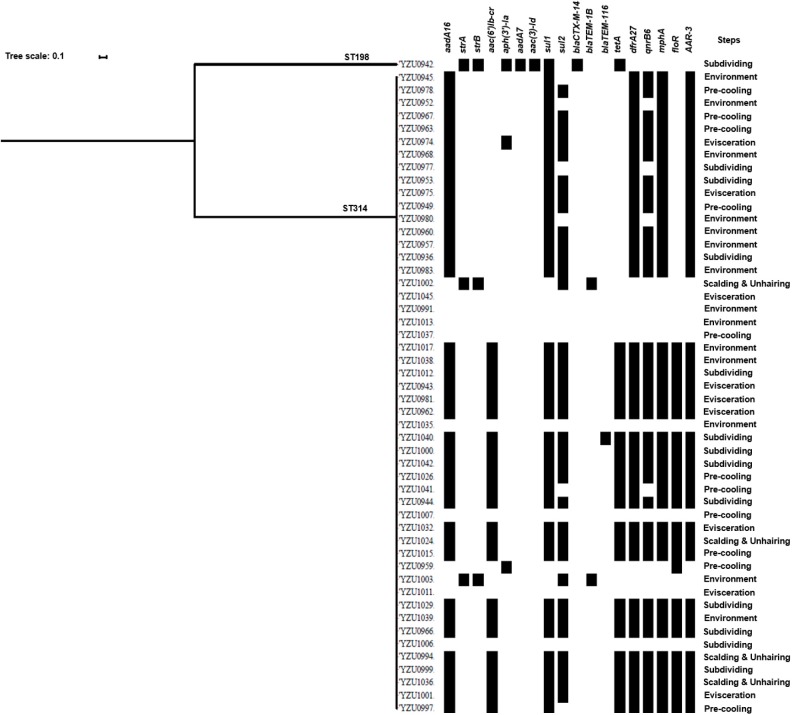
Phylogenetic tree based on core genome and drug resistance genes of *S*. Kentucky. The analysis included 51 *S*. Kentucky isolates from the slaughterhouse of various chicken processing steps. The antimicrobial resistant genes are listed based on the WGS data.

*S*. Kentucky (*n* = 51) was the most predominant serotype isolated in the two visits. The core genome sequence analysis divided the 51 strains into two clusters (Figure 2). Cluster I only contains one strain, while the remaining 50 isolates with the similar core genome sequences belong to cluster II (Figure 2). Interestingly, although only two clusters were shared by these *S*. Kentucky isolates based on the core genome sequences analysis, the antimicrobial resistance gene profiles are diverse in these strains (Figure 2 and Table S6).

By WGS analysis, 18 antimicrobial resistance genes were identified in *S*. Kentucky isolates. The most prevalent antimicrobial resistance genes were *sul1* (78.43%, 40/51), followed by *aadA16* (76.47%, 39/51), *drfA27* (76.47%, 39/51), *mphA* (76.47%, 39/51), *ARR-3* (76.47%, 39/51), and *qnrB6* (68.63%, 35/51). 76.47% of the *S*. Kentucky isolates contained ARGs against five or more types of antibiotics (Table S6), while only eight isolates did not carry any antimicrobial resistance genes. The one *S*. Kentucky ST198 isolate contained the *strA*/*strB*/*aadA7*/*aac(3)-Id, tetA, sul1*, and *blaCTX-M-14* genes, which was very different from *S*. Kentucky ST314 isolates (Figure 2). The AST results confirmed that the *S*. Kentucky ST198 isolate was resistant to tetracycline (*tetA*), sulfamethoxazole (*sul1*), ampicillin/cefazolin/cefotaxime (*blaCTX-M-14*), streptomycin (*strA/B*), and nalidixic acid [*gyrA* (D87Y)]. Thirty-one of 51 *S*. Kentucky isolates were resistant to more than three antimicrobials. Moreover, 20 *S*. Kentucky isolates contained 10 antimicrobial resistance genes, mainly in the genotypes of the *S*. Kentucky that distributed among the four slaughtering stages and environments of the slaughterhouse. The strains carrying seven antimicrobial resistance genes were isolated from the evisceration, pre-cooling, and subdividing stages and environments (Table S6).

### Genomic Analysis of *S*. Enteritidis

**Figure 3:**
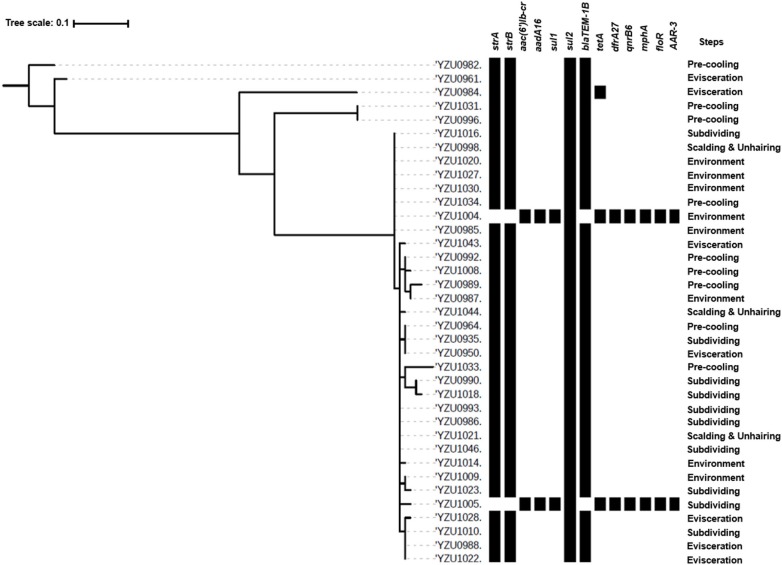
Phylogenetic tree based on core genome and drug resistance genes of *S*. Enteritidis (ST11). The analysis included 37 *S*. Enteritidis isolates from the slaughterhouse of various chicken processing steps. The antimicrobial resistant genes were listed according to the WGS data.

*S*. Enteritidis was identified as another prevalent serotype in the chicken and slaughterhouse, and 37 *S*. Enteritidis isolates were detected in this study. The phylogenetic tree analysis of *S*. Enteritidis isolates was constructed based on the core genome genes, which were divided into five clusters. The main cluster of *S*. Enteritidis contained 32 isolates, while the other clusters contained only one or two isolates (Figure 3). The main cluster of *S*. Enteritidis was detected from all four slaughtering stages and their related environments, while isolates from other clusters were only found at the pre-cooling and evisceration stages.

By WGS analysis, *S*. Enteritidis isolates were divided into three ARG profiles. Even though core genome sequences of *S*. Enteritidis isolates showed diversity, the majority of *S*. Enteritidis showed similar ARG profiles (Figure 3). Thirty-four of 37 *S*. Enteritidis isolates contained a four-ARG profile, which were *sul2, strA/strB*, and *blaTEM-1B*, and these strains were identified through the slaughterhouse (Tables S4, S7). The AST results of these isolates showed that the antimicrobial resistance phenotypes were consistent with the presence of the corresponding resistance genes, including ampicillin (*blaTEM-1B*), streptomycin (*strA/B*), sulfamethoxazole (*sul2*), and nalidixic acid [*gyrA*(D87Y)]. One isolate from the evisceration step contained a very similar ARG profile as the 37 isolates mentioned above with five genes including *sul*2, *str*A/*str*B, *bla*TEM-1B, and *tet*A (Figure 3 and Table S7), and this isolate showed resistance to tetracycline (*tetB*) besides the above antibiotics. Two isolates from subdividing stage and environment, respectively, contained the same ARG profile, which were distinctly different from other *S*. Enteritidis isolates including *aac(6*′*)Ib-cr, aadA16, sul1, sul2, tetA, dfrA27, qnrB6, mphA, floR*, and *AAR-3*. The AST results showed that both isolates were resistant to ciprofloxacin [*qnrB6, aac(6*′*)Ib-cr*], tetracycline (*tetA*), chloramphenicol (*floR*), trimethoprim, sulfamethoxazole (*sul1, sul2*, and *dfrA27*), and nalidixic acid [*gyrA*(D87Y)].

## Discussion

In recent years, the increased prevalence and antimicrobial resistance of *Salmonella* in food has frequently been reported in China, but the prevalence of *Salmonella* in chicken slaughterhouse located in Jiangsu province of China is rarely studied. This study analyzed 200 samples collected from a chicken slaughterhouse in Jiangsu province in 2018 and identified 114 *Salmonella* isolates, with a prevalence rate of 57% (Table 1), which was comparatively high than reported from other studies both globally and domestically. The prevalence rates of *Salmonella* were 30.0 and 9.4% in two different chicken slaughterhouses, respectively, in a study from South Korea (23), while the prevalence rate was 11.1% in a chicken slaughterhouse in the northeast of Algeria (24). A study from Brazil demonstrated that the prevalence of *Salmonella* was only 3.6% in a chicken slaughterhouse (25). In China, the isolation rate of *Salmonella* was 12.7% in chickens in Shandong province (13), while no *Salmonella* was detected in a chicken slaughterhouse in Sichuan province (26). However, in Guangdong province, the prevalence of *Salmonella* in chicken and pork meat at retail markets was 63.6 and 73.1%, respectively, and 62.86% of samples from slaughterhouse were detected to be positive for *Salmonella* (27, 28). In Jiangsu province, the prevalence of *Salmonella* in pig slaughterhouses and retail markets was 71.8 and 70.9%, respectively (20). The isolation rate of *Salmonella* in our study is higher than the previous report in chicken slaughterhouses except that in Guangdong province, but less than that in pig slaughterhouses. These results indicated that the prevalence of *Salmonella* in Jiangsu province was more serious than that in other regions, which increased the potential transmission to humans. These results suggested that the contamination of *Salmonella* in the slaughterhouse should be concerned in control the transmission of *Salmonella*.

Among the various stages in the chicken slaughterhouse, 85 isolates with 17.5, 60.0, 65.0, and 70.0% of *Salmonella* were detected at scalding and dehairing, evisceration, pre-cooling, and subdividing stages, respectively (Table 1). The isolation rates in evisceration, pre-cooling, and subdividing stages were distinctly different from the scalding and dehairing stage, indicating that the evisceration stage was a source for *Salmonella* transmission. Therefore, this step may be the key point for the prevention and control of *Salmonella* contamination in this slaughterhouse. Besides, the isolation rate of *Salmonella* in the environment samples was 72.5%, which was much higher than the previous study with 20% of *Salmonella*-positive environment sample from other chicken slaughterhouses (24). This result demonstrates that the slaughtering environment is another key point for the spread of *Salmonella* in this slaughterhouse.

In total, 114 *Salmonella* isolates were subtyped into six serotypes with *S*. Kentucky and *S*. Enteritidis to be the predominant serotypes in the four slaughtering stages and environments (Figure 1) in the two visits, indicating that *S*. Kentucky and *S*. Enteritidis might be persistent throughout the slaughter line. Moreover, the chickens slaughtered at this abattoir were from different farms. Seven *S*. Corvallis isolates were isolated in the first visit, in which the serotype was also reported in chicken from Brazil with an isolation rate of 7.9% (29). In the present study, the prevalence of *S*. Kentucky and *S*. Enteritidis in the slaughterhouse was 44.7 and 32.5%, which was consistent with findings reported in Guangdong province (27, 28). However, the results were quite different from the results in Sichuan province, in which *S*. Derby and *S*. Typhimurium were identified as the most common serotypes (26). *S*. Enteritidis was reported as the most common serotype in human cases, which was mainly detected from laying hens, followed by broiler meat (4). *S*. Enteritidis was also the most common serotype of human *Salmonella* infections in the USA during 2011 and 2016 (3). In China, *S*. Enteritidis was recognized as the most frequently isolated *Salmonella* serotype in chicken meat (30, 31). The above data indicated that the *S*. Enteritidis was recognized as a dominant serotype worldwide. The most common ST of *S*. Enteritidis was ST11 in Hubei, Shanghai, and Shandong province, China, which was consistent with our study (32–34). In addition, the ST11 was also identified as the predominant ST of *S*. Enteritidis in Iran, Brazil, Denmark, Japan, and USA, indicating that the ST11 is probably an ancestral clone of *S*. Enteritidis successfully scattered in all of these geographically diverse countries (35).

*S*. Kentucky was identified as the most common serotype in this study (Table 1). Previous studies indicated that *S*. Kentucky was mainly found in North America, but that the isolation rate of *S*. Kentucky in retail meat was significantly increasing in China (27, 36). Human infection cases by *S*. Kentucky were reported in Europe and USA, and *S*. Kentucky was the seventh top serotype-causing human salmonellosis in Europe during 2017 (3, 4). ST314 (53/54) was predominant in the *S*. Kentucky isolates, while only one isolate belonged to ST198 (1/54) in this study. The most common ST of *S*. Kentucky isolates from Hubei province of China was ST314, while most of the isolates from Shandong province were ST198 (32, 34). Furthermore, ST198 was the most common clone among the *S*. Kentucky isolates from chicken in Vietnam and humans in USA (37, 38). Besides, the ST198 was considered as a worldwide-disseminated multidrug-resistant clone, which may originate outside of the North America (38), and our study also showed that the ST198 isolates could resistance to tetracycline, sulfamethoxazole, ampicillin/cefazolin/cefotaxime, streptomycin and nalidixic acid. By now, studies about the prevalence of *S*. Kentucky in chicken was limited and no infection casein humans was reported in China. However, our studies showed that the prevalence of *S*. Kentucky in chicken carcass was increasing, which indicated a potential risk of transmitting it to the public by the food chain in China. Further studies are required to explore the relationship between the recent and early isolates of *S*. Kentucky in China.

The antimicrobial resistance in *Salmonella* is one of the main concerns of its infection in humans. This study analyzed genotypes of antimicrobial resistance genes presenting in all 114 *Salmonella* isolates, which showed diverse relationship to the different serotypes. Based on the core genome analysis, the most prevalent serotype *S*. Kentucky was only divided into two clusters with a predominant cluster containing 51 isolates and one isolate to the other cluster. By correlating the core genome to the genotypes of antibiotic resistance genes, we observed a high diversity of the antibiotic resistance genes in the predominant cluster of *S*. Kentucky isolates (Figure 2 and Table S6), indicating that the multidrug resistance of *S*. Kentucky was less related to the core genome. Previous studies showed that *S*. Kentucky were multidrug-resistance serotypes (38–41), while *S*. Kentucky isolates in this study contained antibiotic resistance gene from more than five different antibiotic groups. *S*. Enteritidis isolates in this study showed a close relationship of the core genome clusters to the genotypes of its antibiotic resistance genes (Figure 3 and Table S7). Three types of the antimicrobial resistance genes of *S*. Enteritidis were identified, including the aminoglycoside resistance genes *strA*/*strB*, sulfonamide resistance gene *sul2*, and β-lactam resistance gene *blaTEM-1B*. These four genes were located in the IncX1 plasmid, which was predominant in *S*. Enteritidis. The IncX1 plasmid may mediate resistance genes transmission of *S*. Enteritidis in this slaughterhouse. Of 37 *S*. Enteritidis isolates, 35 contained the point mutant in *gyrA* gene for nalidixic acid resistance. A previous study showed that *S*. Enteritidis were highly resistant to nalidixic acid (91.3%), ampicillin (39.13%), and streptomycin (28.70%) in Jiangsu province, China (42), which were confirmed with our antimicrobial genotype analysis. Moreover, a study from Thailand also demonstrated similar results, in which *S*. Enteritidis showed highest resistance rates to nalidixic acid (83.2%) and ampicillin (50.05%) (43). A previous study showed that aminoglycoside resistance genes *aadA5, aadA7*, and *aac(3)-Id*, and trimethoprim resistance genes *drfA14* and *drfA17* were only detected in isolates from human infection cases (44). However, these genes were also observed in our *Salmonella* isolates from chicken carcasses and the slaughter environments, indicating that these multidrug-resistant *Salmonella* isolates might have the risk to transmit from chicken meat to humans.

The predominant serotypes of *Salmonella* isolated from the food handlers' fecal matter in Jiangsu province, China, were *S*. Typhimurium (16.1%), followed by *S*. Derby (13.5%), *S*. Enteritidis (11.4%), and *S*. London (11.4%) (45). The high prevalence of *S*. Enteritidis in humans may be caused by chicken meat (46). Multidrug resistance rate among the strains was 73.4%, and the predominant phenotype among the MDR was Amp, Sul, and Tet resistance (47); we also found the genes responsible for these antibiotic resistance in this study, indicating the transmission of *Salmonella* from chicken to humans. Compared with the *Salmonella* isolated from humans in Hubei, Guangdong, and Zhejiang province of China, the *S*. Enteritidis was the common predominant serotype, indicating that the prevalence of *S*. Enteritidis was serious in Chinese people (32, 48, 49). Besides, almost all of the *S*. Enteritidis were multidrug resistance. The most common phenotypes of antimicrobial resistance in *S*. Enteritidis from Zhejiang province were nalidixic acid, sulfonamides, ampicillin, and streptomycin, and similar phenotypes were identified in Hubei and Guangdong province, which was consistent with our genotypes of AGRs in *S*. Enteritidis (32, 48, 49). These results indicate that these multidrug-resistant *Salmonella* isolates could be potentially transmitted from chicken meat to humans. This study calls for further attention in the prevention and control of foodborne disease caused by *Salmonella*, as well as improvement in the environment of food slaughterhouses.

## Conclusions

This study investigated the overall prevalence of *Salmonella* in a chicken slaughterhouse in Jiangsu province of China. By WGS, serotypes and MLST types of all *Salmonella* isolates were analyzed, and *S*. Kentucky and *S*. Enteritidis were observed as the predominant serotypes in the slaughter line and environment. Meanwhile, a high prevalence of multidrug-resistant *Salmonella* was observed in chicken carcasses from all slaughtering steps and environment, indicating a potential risk transmission from chicken slaughterhouse to humans. Further studies will be needed to elucidate the extent to which human infections are caused by the *Salmonella* contamination from chicken slaughtering.

## Data Availability Statement

The datasets generated for this study can be found in the European Nucleotide Archive, accession number PRJEB34962: https://www.ebi.ac.uk/ena.

## Ethics Statement

This study was carried out in accordance with the principles of the Basel Declaration and recommendations of the institutional administrative committee and ethics committee of laboratory animals, Animal Welfare and Ethics Committees of Yangzhou University. The protocol was approved by the Animal Welfare and Ethics Committees of Yangzhou University.

## Author Contributions

DG, YT, CM, and ZP contributed to the conception and design of this study. DG, ZW, and XC were responsible for the acquisition of the data analyzed in this study. DG, XK, ZP, and XJ were involved in the analysis and interpretation associated with this work. All the authors were involved in manuscript revisions and final approval of the version to be published.

### Conflict of Interest

The authors declare that the research was conducted in the absence of any commercial or financial relationships that could be construed as a potential conflict of interest.

## Supplementary Material

Figure S1 Verification of *Salmonella* isolates by *stn* PCR. Lane 1 to 26 represent the PCR results of potential *Salmonella* isolates randomly picked single colonies that grew on the XLT4 agar plate. Lane M was DL2, 000DNA marker (Takara, Japan), the negative control used was *E. coli* DH5a, and the positive control was *Salmonella* Typhimurium LT2. PCR products were separated on a 1% agarose gel and stained with ethidium bromide.

Table S1 The assembly information of whole-genome sequencing.

Table S2 The analysis information of whole-genome sequencing.

Table S3 Plasmid replicons.

Table S4 Antimicrobial resistance genes of the *Salmonella* isolates.

Table S5 Mutation of the QRDRs in different serotypes.

Table S6 Antimicrobial resistant genes of *S*. Kentucky.

Table S7 Antimicrobial resistant genes of *S*. Enteritidis.
