# Supplementary material for: Prevalence of Salmonella Isolates and Their Distribution Based on Whole-Genome Sequence in a Chicken Slaughterhouse in Jiangsu, China
Source: Front Vet Sci. 2020 Feb 21;7:29. doi: 10.3389/fvets.2020.00029 (PMC7046563; doi:10.3389/fvets.2020.00029)
Supplement: Figure S1 — Verification of Salmonella isolates by stn PCR. Lane 1 to 26 represent the PCR results of potential Salmonella isolates randomly picked single colonies that grew on the XLT4 agar plate. Lane M was DL2, 000DNA marker (Takara, Japan), the negative control used was E. coli DH5a, and the positive control was Salmonella Typhimurium LT2. PCR products were separated on a 1% agarose gel and stained with ethidium bromide. [file Data_Sheet_1.docx]

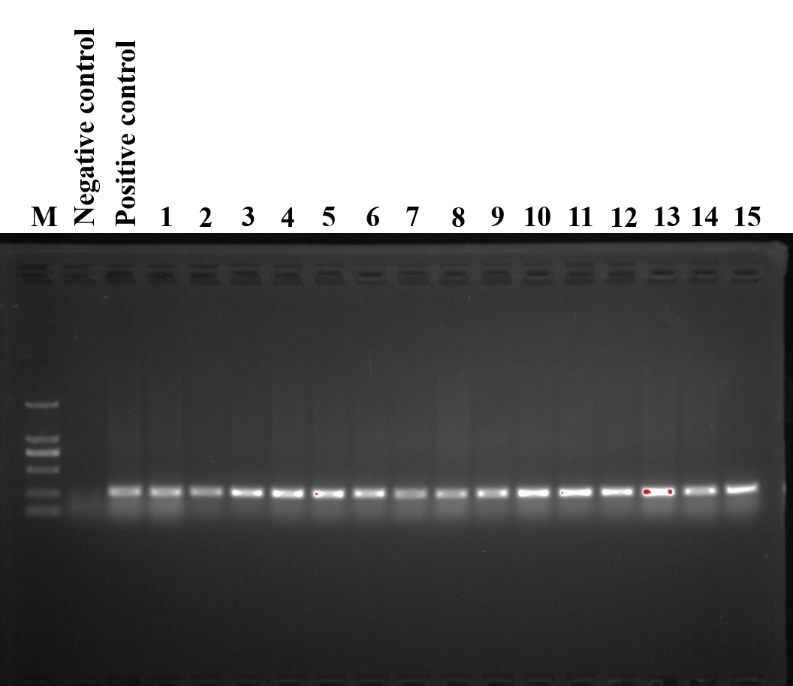


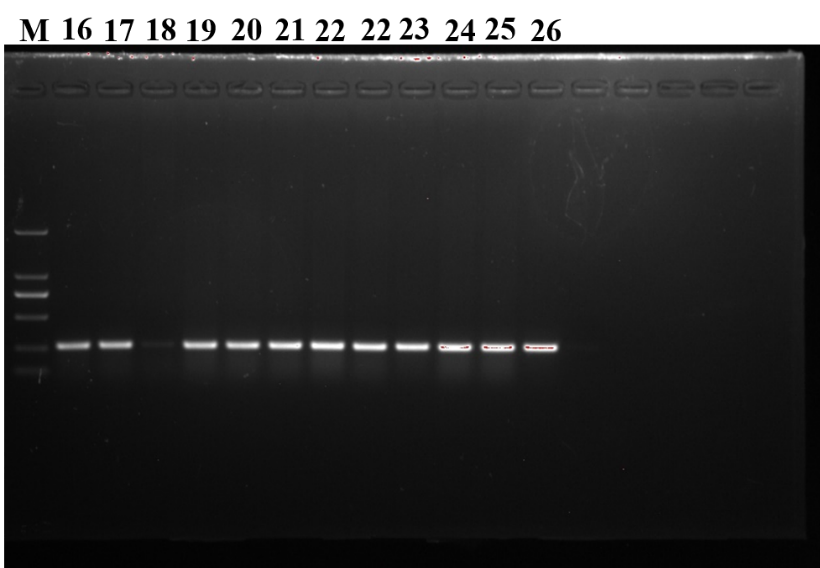


**Figure S1. Verification of *Salmonella* isolates by *stn* PCR. Lane 1 to 26 represent the PCR results of potential *Salmonella* isolates randomly picked single colonies that grew on the XLT4 agar plate. Lane M was DL2,000 DNA marker (Takara, Japan), the negative control used was *E. coli* DH5a, and the positive control was *Salmonella* Typhimurium LT2. PCR products were separated on a 1% agarose gel and stained with ethidium bromide.**

**Table S3. Plasmid replicons**

| **Serotypes** | **Plasmid name** | **# of isolates harboring the plasmid/ total isolates (%)** |
| --- | --- | --- |
| ***S*. Enteritidis** | IncFIB(S) | 32/114 (28.1%) |
| **(n=37)** | IncFII(S) | 32/114 (28.1%) |
|  | IncX1 | 37/114 (32.5%) |
|  | IncI1 | 2/114 (17.5%) |
| ***S*. Kentucky** | IncR | 42/114 (36.8%) |
| **(n=51)** | IncX1 | 16/114 (14.0%) |
|  | IncI1 | 3/114 (2.6%) |
|  | IncQ1 | 1/114 (0.9%) |
|  | none | 8/114 (7.0%) |
| ***S*. Indiana** | IncHI2 | 3/114 (2.6%) |
| **(n=15)** | IncN | 3/114 (2.6%) |
|  | IncQ1 | 3/114 (2.6%) |
|  | IncX1 | 2/114 (1.8%) |
|  | p0111 | 2/114 (1.8%) |
|  | Col440I | 1/114 (0.9%) |
|  | IncHI2A | 1/114 (0.9%) |
|  | none | 12/114 (10.5%) |
| ***S*. Corvallis** | Col440I | 7/114 (6.1%) |
| **(n=7)** | IncQ1 | 7/114 (6.1%) |
| ***S*. Hadar**  **(n=1)** | IncFII(p96A) | 1/114 (0.9%) |
| **I 4,[5],12:i:-** | IncQ1 | 3/114 (2.6%) |
| **(n=3)** | IncFIA(HI1) | 1/114 (0.9%) |
|  | IncR | 1/114 (0.9%) |
|  | IncHI2 | 1/114 (0.9%) |
|  | IncHI2A | 1/114 (0.9%) |
|  | Col440I | 1/114 (0.9%) |
|  | IncI1 | 1/114 (0.9%) |

**Table S4. Antimicrobial resistance genes of the *Salmonella* isolates**

| **Antibiotic** | **Genes** | **Numbers (n) in different serotypes** | | | | | | **Percent (%)** |
| --- | --- | --- | --- | --- | --- | --- | --- | --- |
|  |  | ***S*. Indiana**  **(15)** | ***S*. Kentucky**  **(51)** | ***S*. Enteritidis**  **(37)** | ***S*. Corvallis**  **(7)** | ***S*. Hadar**  **(1)** | **I 4,[5],12:i:-**  **(3)** |  |
| **Aminoglycoside** | *strA* | 15 | 3 | 35 | 3 | 1 | 3 | 60 (52.63) |
|  | *strB* | 15 | 3 | 35 | 3 | 1 | 3 | 60 (52.63) |
|  | *aadA1* |  |  |  |  |  | 1 | 1 (0.87) |
|  | *aadA2* |  |  |  |  |  | 1 | 1 (0.87) |
|  | *aadA5* | 14 |  |  |  |  |  | 14 (12.28) |
|  | *aadA7* |  | 1 |  |  |  |  | 1 (0.87) |
|  | *aadA16* |  | 39 | 2 |  |  | 1 | 42 (36.84) |
|  | *aph(3')-IIa* | 2 |  |  |  |  |  | 2 (1.75) |
|  | *aph(3')-Ic* |  | 1 |  |  |  |  | 1 (0.87) |
|  | *aph(3')-Ia* |  | 2 |  | 2 |  |  | 2 (1.75) |
|  | *aph(4)-Ia* | 14 |  |  |  |  |  | 14 (12.28) |
|  | *aac(3)-Id* |  | 1 |  |  |  |  | 1 (0.87) |
|  | *aac(3)-IVa* | 14 |  |  |  |  |  | 14 (12.28) |
|  | *aac(6')Ib-cr* | 14 | 23 | 2 |  |  |  | 39 (34.21) |
|  | *armA* | 2 |  |  |  |  |  | 2 (1.75) |
|  | *rmtB* | 1 |  |  |  |  |  | 1 (0.87) |
| **Sulphonamide** | *sul1* | 11 | 40 | 2 |  |  | 1 | 54 (47.37) |
|  | *sul2* | 2 | 35 | 37 | 3 |  | 3 | 80 (70.18) |
|  | *sul3* |  |  |  |  |  | 1 | 1 (0.87) |
| **β-lactam** | *blaCTX-M-55* | 12 |  |  |  |  |  | 12 (10.53) |
|  | *blaCTX-M-65* | 2 |  |  |  |  | 2 | 4 (3.51) |
|  | *blaCTX-M-14* |  | 1 |  |  |  |  | 1 (0.87) |
|  | *blaOXA-1* | 13 |  |  |  |  |  | 13 (11.40) |
|  | *blaOXA-10* |  |  |  |  |  | 2 | 2 (1.75) |
|  | *blaTEM-1B* | 2 | 2 | 35 |  |  | 1 | 40 (35.09) |
|  | *blaTEM-116* |  | 1 |  |  |  |  | 1 (0.87) |
| **Tetracycline** | *tetA* | 15 | 24 | 3 | 3 | 1 |  | 46 (40.35) |
|  | *tetB* |  |  |  |  |  | 1 | 1 (0.87) |
| **Trimethoprim** | *dfrA14* |  |  |  |  |  | 2 | 2 (1.75) |
|  | *dfrA17* | 14 |  |  |  |  |  | 14 (12.28) |
|  | *dfrA27* |  | 39 | 2 |  |  | 1 | 43 (37.72) |
| **Quinolone** | *oqxA* | 2 |  |  |  |  |  | 2 (1.75) |
|  | *oqxB* | 3 |  |  |  |  |  | 3 (2.63) |
|  | *qnrS1* |  |  |  | 6 |  | 2 | 8 (7.02) |
|  | *qnrB6* |  | 35 | 2 |  |  | 1 | 38 (33.33) |
| **Macrolide** | *mphA* | 3 | 39 | 2 |  |  |  | 44 (38.60) |
| **Phenicol** | *floR* | 15 | 24 | 2 | 7 |  | 2 | 50 (43.86) |
|  | *catB3* | 14 |  |  |  |  |  | 14 (12.28) |
| **Rifampicin** | *ARR-3* | 14 | 39 | 2 |  |  | 3 | 58 (50.88) |
| **Fosfomycin** | *fosA* | 2 |  |  |  |  |  | 2 (1.75) |

**Table S5. Mutation of the QRDRs in different serotypes**

| **Serotypes** | **Number** | ***gyrA*** | ***gyrB*** | ***parC*** | ***parE*** |
| --- | --- | --- | --- | --- | --- |
| ***S*. Indiana** | 15 | S83F, D87N |  | T57S, S80R, S395N, A469S, T620A, A628S |  |
| ***S*. Kentucky** | 50 |  |  | T57S, A469S, T620A |  |
|  | 1 | S83F, D87G |  | T57S, S80I, S395N, A469S, T620A |  |
| ***S*. Enteritidis** | 35 | D87Y |  | S255T, S395N, A469S, T620A |  |
| ***S*. Corvallis** | 7 |  |  | T57S, T620A | Q135R |
| ***S*. Hadar** | 1 | S83F |  | T57S, T620A |  |
| **I 4,[5],12:i:-** | 3 |  |  |  |  |

**Table S6. Antimicrobial resistant genes of *S.* Kentucky**

| **Steps (numbers)** | | **Antimicrobial resistant genes (numbers)** | **Total** |
| --- | --- | --- | --- |
| **Scalding & Unhairing (4)** | | *strA*/*strB/sul2/blaTEM-1B* (4) | 1 |
|  | | *aadA16/aac(6')Ib-cr/sul1/sul2/tetA/dfrA27/qnrB6/mphA/floR/ARR-3* (10) | 3 |
| **Evisceration (9)** | | - | 2 |
|  | | *aadA16/sul1/sul2/dfrA27/qnrB6/mphA/ARR-3* (7) | 1 |
|  | | *aadA16/aph(3')-Ia/sul1/sul2/dfrA27/qnrB6/mphA/ARR-3* (8) | 1 |
|  | | *aadA16/aac(6')Ib-cr/sul1/sul2/tetA/dfrA27/qnrB6/mphA/floR/ARR-3* (10) | 5 |
| **Pre-cooling (11)** | | - | 2 |
|  | | *aph(3')-Ia/floR* (2) | 1 |
|  | | *aadA16/sul1/sul2/dfrA27/qnrB6/mphA/ARR-3* (7) | 4 |
|  | | *aadA16/aac(6')Ib-cr/sul1/tetA/dfrA27/mphA/floR/ARR-3* (8) | 1 |
|  | | *aadA16/aac(6')Ib-cr/sul1/tetA/dfrA27/qnrB6/mphA/floR/ARR-3* (9) | 1 |
|  | | *aadA16/aac(6')Ib-cr/sul1/sul2/tetA/dfrA27/qnrB6/mphA/floR/ARR-3* (10) | 2 |
| **Subdividing (13)** | | - | 1 |
|  | | *aadA16/sul1/dfrA27/qnrB6/ARR-3* (5) | 1 |
|  | | *aadA16/sul1/sul2/dfrA27/qnrB6/mphA/ARR-3* (7) | 2 |
|  | | *strA*/*strB/aph(3')-Ia/aadA7/aac(3)-Id/sul1/blaCTX-M-14/tetA* (8) | 1 |
|  | | *aadA16/aac(6')Ib-cr/sul1/sul2/tetA/dfrA27/qnrB6/mphA/floR/ARR-3* (10) | 7 |
|  | | *aadA16/aac(6')Ib-cr/sul1/sul2/blaTEM-116tetA/dfrA27/qnrB6/mphA/floR/ARR-3* (11) | 1 |
| **Environment (14)** | | - | 3 |
|  | | *strA*/*strB/sul2/blaTEM-1B* (4) | 1 |
|  | | *aadA16/sul1/dfrA27/mphA/ARR-3* (5) | 2 |
|  | | *aadA16/sul1/dfrA27/qnrB6/mphA/ARR-3* (6) | 1 |
|  | | *aadA16/sul1/sul2/dfrA27/qnrB6/mphA/ARR-3* (7) | 4 |
|  | | *aadA16/aac(6')Ib-cr/sul1/sul2/tetA/dfrA27/qnrB6/mphA/floR/ARR-3* (10) | 3 |
| **Total** |  |  | 51 |

**Table S7. Antimicrobial resistant genes of *S.* Enteritidis**

| **Steps (numbers)** | | **Antimicrobial resistant genes (numbers)** | **Total** |
| --- | --- | --- | --- |
| **Scalding & Unhairing (3)** | | *strA*/*strB/sul2/blaTEM-1B* (4) | 3 |
| **Evisceration (7)** | | *strA*/*strB/sul2/blaTEM-1B* (4) | 6 |
|  | | *strA*/*strB/sul2/blaTEM-1B/tetA* (5) | 1 |
| **Pre-cooling (9)** | | *strA*/*strB/sul2/blaTEM-1B* (4) | 9 |
| **Subdividing (10)** | | *strA*/*strB/sul2/blaTEM-1B* (4) | 9 |
|  | | *aac(6')Ib-cr/aadA16/sul1/sul2/tetA/dfrA27/qnrB6/mphA/floR/ARR-3* (10) | 1 |
| **Environment (8)** | | *strA*/*strB/sul2/blaTEM-1B* (4) | 7 |
|  | | *aac(6')Ib-cr/aadA16/sul1/sul2/tetA/dfrA27/qnrB6/mphA/floR/ARR-3* (10) | 1 |
| **Total** |  |  | 37 |
